# Supplementary material for: Overexpression of a Voltage-Dependent Anion-Selective Channel (VDAC) Protein-Encoding Gene, MsVDAC, from Medicago sativa Confers Cold and Drought Tolerance to Transgenic Tobacco
Source: Genes (Basel). 2021 Oct 27;12(11):1706. doi: 10.3390/genes12111706 (PMC8617925; doi:10.3390/genes12111706)
Supplement: Supplementary file 1 [file genes-12-01706-s001.zip › Tables S1 and S2.pdf]

Table S1. qPCR Primers of stress response related genes.

Gene Name	Primer Sequence	Gene Function
MsActin-F	TTTGAGACTTTCAATGTGCCCGCC	Action gene of alfalfa
MsActin-R	TAGCATGTGGGAGTGCATAACCCT	
NtActin-F	GCTTTCTTCGTCCCATCA	Action gene of tobacco
NtActin-R	CCCCAAGTACCCTCGTAT	
CBL1-F	GCCTCACAAGAAGAGAAGATCA	calcineurin B-like protein 1
CBL1-R	AACTTCATCTCAGACTCGCATAG	
GAPC-F	GTGCTGATTTCGTTGTGGAATC	glyceraldehyde-3-phosphate
GAPC-R	GAGCAGAGATCACAACCTTCTT	dehydrogenase, cytosolic
BI-1-F	CTTTGGAACATTGGTGGCTTAC	bax inhibitor 1-like
BI-1-R	CCATCAGAAGTGCTATCCTCTTT	
Hxk3-F	TTCATCAACCTCCTGGTAAGC	hexokinase-2-like
Hxk3-R	CCTTTCGTCCACCTGATAAGAG	
GR1-F	CAGAATGACCAGAGTCCTCTAAC	protein gamma response 1
GR1-R	CCAGCACTTCATGTTTGTCTTC	
Cu,Zn-SOD-F	AGCAGCAGTGAAGGTGTTAG	Superoxide dismutase [Cu-Zn] 2
Cu,Zn-SOD-R	GGCCAGAGACATTTCCAGTAA	
MnSOD-F	CGGCAATTAGCGGTGACATA	superoxide dismutase [Mn],
MnSOD-R	ATGGCGTCATGTAGCTGTTC	mitochondrial-like
Ltp1-F	CGAATTTGGCACCTTGTCTTG	non-specific lipid-transfer
Ltp1-R	GCAGAATTCACCAGAGCCTTA	protein 1
ERD10B-F	CAAGGCGGAAGAAGGAAGAA	dehydrin DHN1-like
ERD10B-R	CGTAGTTGTTGCAGTTGAATGAG	
SnRK2-F	CTGTAGGGACGCCTGCTTATG	serine/threonine-protein kinase
SnRK2-R	TGGACTTGTTCGGGGATTGA	SRK2A

Table S2. The full-length cDNA of *MsVDAC*.

Gene	Sequence
MsVDAC	ATGGCTAACGGTCCAGCACCGTTTTCTGAGATTGGCAAAAGAGCAAG<br>AGACCTCTTGTACAAGGATTATAATTTCGACCACAAGTTTAGCCTGTCGAT<br>TCCAAGTTCCACTGGATTGGGCCTTACAGCTACAGGGTTGAAGAGGGATA<br>AATTTTTTGTTGGTGACTTAAACACGCTTTACAAGAGTGGAAATGTTACAG<br>TGGATGTGAAAGTCAATACAGACTCTAATGTATCTACAAAAGTGACTTTGA<br>ATGATGTTTCCCTTTCCCATAGTAAAAAAGTAGCGTTGAGCTTCAATATAC<br>CTGATCACAAGTCAGGAAAGTTGGATGTGCAGTATCTTCATCCTCATGCAG<br>CTATTGATTCCAGTATTGGCTTGAACCCAGCCCCTAAATTGGAGCTTTCCG<br>CTGCAATTGGAAGCAAAGATATATCTATGGGTGCTGAAGTTGGATTCGATA<br>CAACCTCTGCTTCATTCTCAACATATAATGCCGGGATTGCCTTCAATAAAC<br>CAGATTTCTCTGCAACACTTATGCTGGCTGATAAAGGACAGTCTCTGAAG<br>GCATCTTACATTCATTATGTGGACCGCCCAGATGGATTAACAGTTGCTGCT<br>GAACTCGCTCACAAGTTGTCCTCTTCTGAGAACAGATTTACCTTTGGGAC<br>CTCACAGTCAATAGATCCAAAAACAGTTTTGAAGACTCGATTCAGTGATG<br>ATGGCAAAGCTGCCTTCCAATGCCAACGAGCGTGGAGGCCAAATTCACTC<br>ATAACCCTATCTGCCGAGTATGACTCCAAAAAAATCATTGGTTCACCTGCC<br>AAATTCGGTCTCGCTCTTTCTCTCAAGCCTTAA
