# Supplementary figures and images for: Overexpression of a Voltage-Dependent Anion-Selective Channel (VDAC) Protein-Encoding Gene, MsVDAC, from Medicago sativa Confers Cold and Drought Tolerance to Transgenic Tobacco
Source: Genes (Basel). 2021 Oct 27;12(11):1706. doi: 10.3390/genes12111706 (PMC8617925; doi:10.3390/genes12111706)

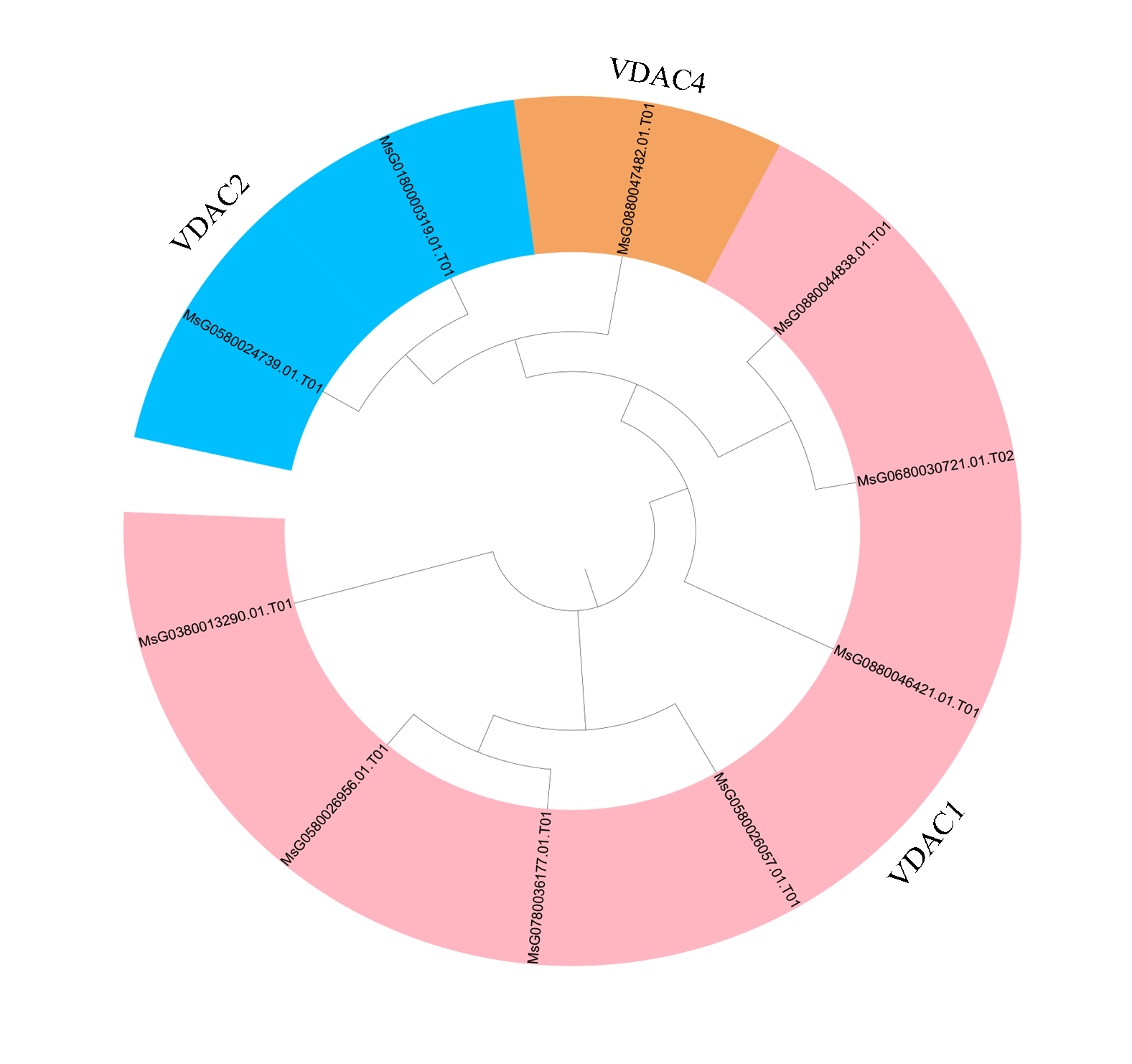

Supplement: Supplementary file 1 [file genes-12-01706-s001.zip › Figure S1.tif]

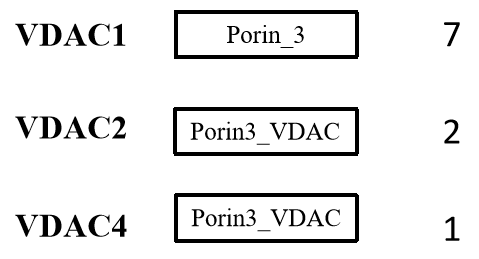

Supplement: Supplementary file 1 [file genes-12-01706-s001.zip › Figure S2.tif]

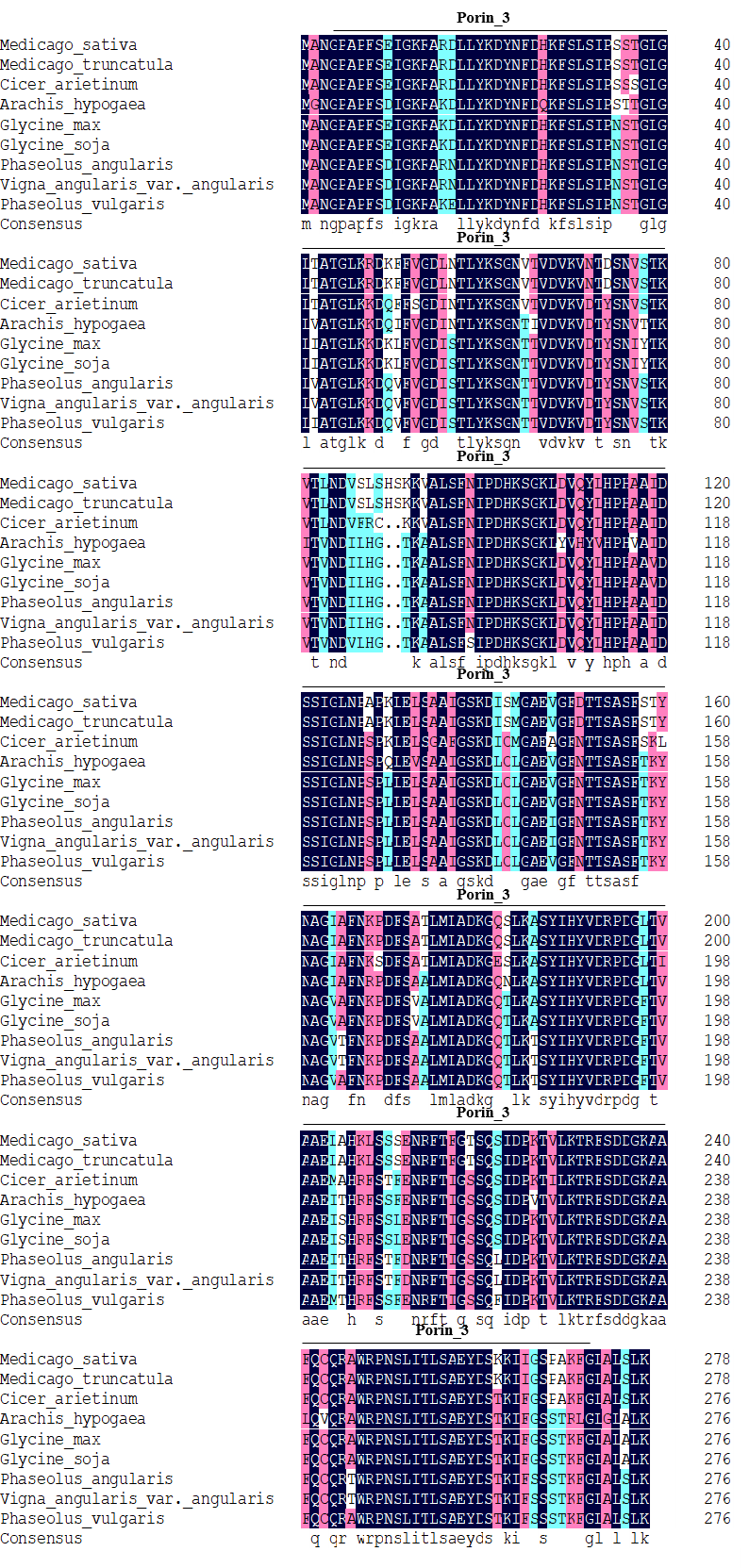

Supplement: Supplementary file 1 [file genes-12-01706-s001.zip › Figure S3.tif]

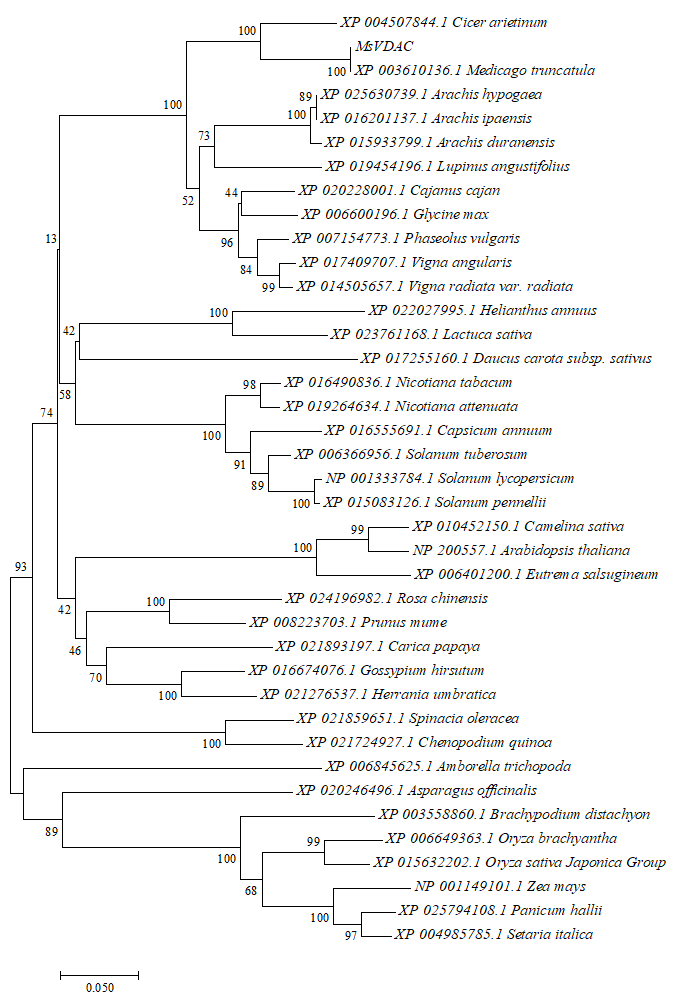

Supplement: Supplementary file 1 [file genes-12-01706-s001.zip › Figure S4.tif]

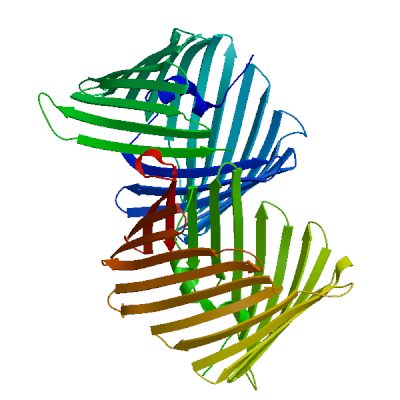

Supplement: Supplementary file 1 [file genes-12-01706-s001.zip › Figure S5.png]

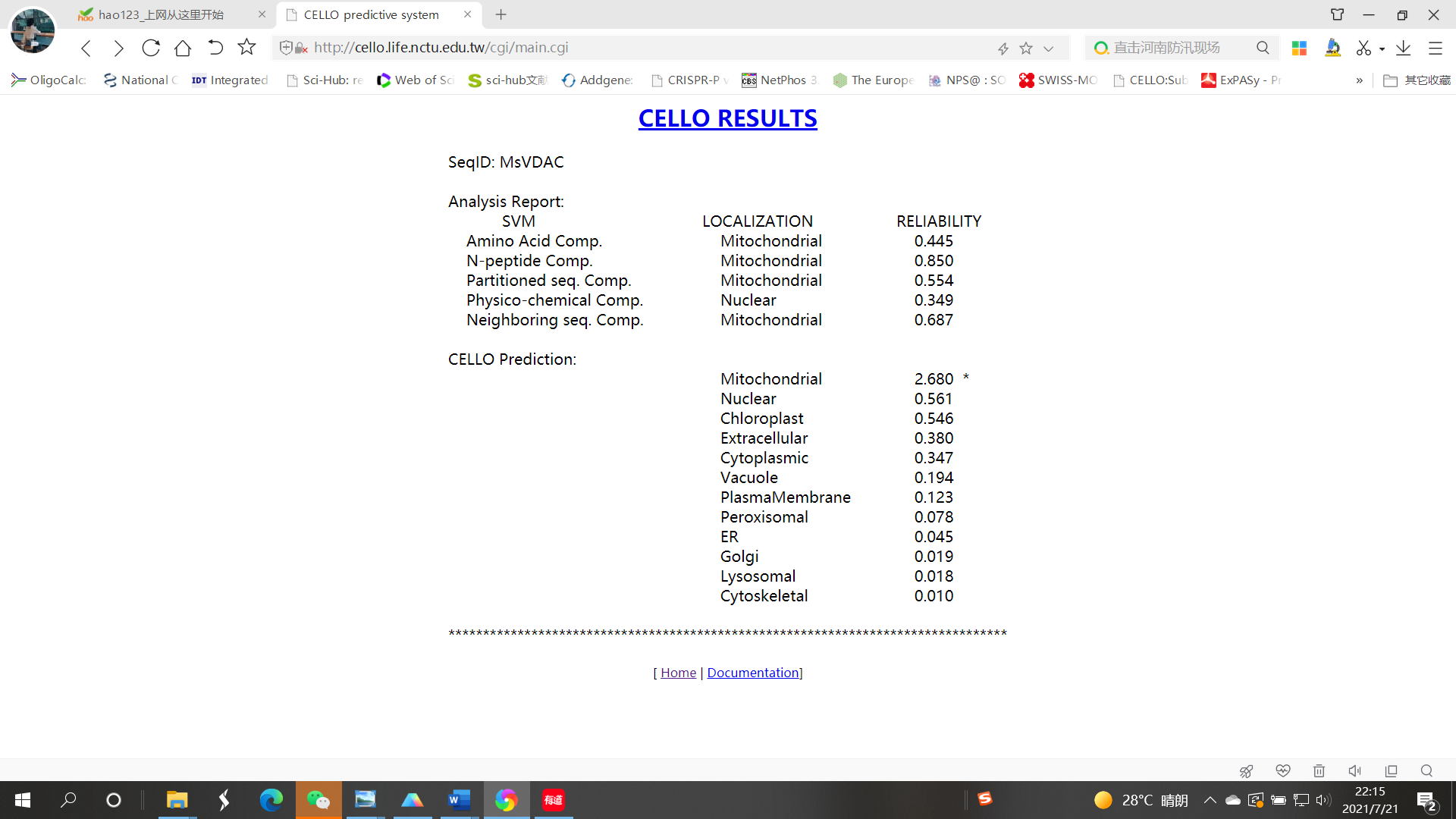

Supplement: Supplementary file 1 [file genes-12-01706-s001.zip › Figure S6.png]
